# Multifaceted taxonomy of two *Dactylogyrus* species on *Enteromius paludinosus*: Integrating light microscopy, scanning electron microscopy and molecular approaches

**DOI:** 10.1051/parasite/2024077

**Published:** 2025-01-30

**Authors:** Mpho Maduenyane, Quinton Marco Dos Santos, Annemariè Avenant-Oldewage

**Affiliations:** Department of Zoology, University of Johannesburg Auckland Park P.O. Box 524 Johannesburg South Africa

**Keywords:** Dactylogyridae, DNA Barcoding, Sclerite morphology, Monogenea, Monopisthocotylea, Life below water

## Abstract

*Dactylogyrus* Diesing, 1850 is the most speciose genus of platyhelminths with more than 900 species, and over a hundred species recorded from Africa. Of the latter, six are from the straightfin barb, *Enteromius paludinosus* (Peters)*. Dactylogyrus teresae* Mashego, 1983 and *Dactylogyrus dominici* Mashego, 1983 were collected from *E. paludinosus* in the Vaal River system, Gauteng, South Africa and their taxonomic data revised using standard protocols and modern approaches, alongside the type material. Whole worms were mounted on glass slides with glycerine ammonium picrate (GAP) and studied using light microscopy (LM). For scanning electron microscopy (SEM), whole worms were placed on concavity slides and the soft tissue digested to release the sclerotised copulatory organs and haptoral sclerites. A combination of these approaches (LM and SEM) was employed for the first time to study the sclerotised structures of GAP-mounted material. Soft tissues of SEM analysed specimens were genetically characterised using CO1 mtDNA, 18S-ITS1-5.8S rDNA and partial 28S rDNA fragments. Phylogenetic topologies were constructed using Bayesian inference. Results confirmed the morphologic and genetic distinctness of *D. dominici* and *D. teresae,* highlighting the importance of studying the varying orientations of specifically the vagina and transverse bar. This study presents a new locality record, the first SEM study of isolated sclerotised structures, as well as the first molecular data for the *Dactylogyrus afrobarbae*-like species. The multifaceted approaches applied to the same specimen in this study enabled improved resolution of individual specimens, showing promise for studies where limited specimens are available.

## Abstract (fr)

*Dactylogyrus* Diesing, 1850 est le genre de Plathelminthes qui a le plus d’espèces (plus de 900) dont plus d’une centaine recensées en Afrique. Parmi ces dernières, six proviennent du barbu *Enteromius paludinosus* (Peters). *Dactylogyrus teresae* Mashego, 1983 et *Dactylogyrus dominici* Mashego, 1983 ont été collectés chez *E. paludinosus* dans le système fluvial de Vaal, Gauteng, Afrique du Sud et leurs données taxonomiques ont été révisées à l’aide de protocoles standard et d’approches modernes, parallèlement au matériel type. Les vers entiers ont été montés sur des lames de verre avec du picrate d’ammonium glycériné (PAG) et étudiés à l’aide d’un microscope optique (MO). Pour la microscopie électronique à balayage (MEB), des vers entiers ont été placés sur des lames à concavité et les tissus mous ont été digérés pour libérer les organes copulateurs sclérifiés et les sclérites haptoraux. Une combinaison de ces approches (MO et MEB) a été utilisée pour la première fois pour étudier les structures sclérifiées du matériel monté en PAG. Les tissus mous des spécimens analysés par MEB ont été caractérisés génétiquement à l’aide de fragments d’ADNmt CO1, d’ADNr 18S-ITS1-5,8S et d’ADNr 28S partiel. Les topologies phylogénétiques ont été construites à l’aide de l’inférence bayésienne. Les résultats ont confirmé la distinction morphologique et génétique de *D. dominici* et de *D. teresae*, soulignant l’importance d’étudier en particulier les différentes orientations du vagin et de la barre transversale. Cette étude présente un nouvel enregistrement de localité, la première étude MEB de structures sclérifiées isolées, ainsi que les premières données moléculaires pour les espèces de type *Dactylogyrus afrobarbae*. Les approches multidimensionnelles appliquées au même échantillon dans cette étude ont permis d’améliorer la résolution des échantillons individuels, ce qui s’avère prometteur pour les études où les échantillons disponibles sont limités.

## Introduction

The straightfin barb, *Enteromius paludinosus* (Peters, 1852) is distributed across African freshwater systems [23, 66]. This fish species has been reported to host a wide variety of ecto- and endoparasites [56]. One of the monogenean groups infecting this fish species is *Dactylogyrus* (Diesing, 1850), which comprises oviparous ectoparasites that primarily infect cyprinoid fishes [12, 13, 47–51]. According to Gibson *et al.* [22] and many other recent descriptions, *e.g.*, [1, 20, 37, 52, 53], *Dactylogyrus* is the most speciose genus of Platyhelminthes Minot, 1876 with over 900 nominal species. Moreover, dactylogyrids tend to exhibit varying degrees of host specificity to fishes of the Cypriniformes Bleeker, 1859 with some species being strictly specific to a single host species, while others are less specific [12, 49, 60]. As the majority of dactylogyrids exclusively parasitise cypriniform fishes, the number of nominal species in this taxon is estimated to be even higher than what is currently reported, considering that many cypriniform hosts have not been surveyed for ectoparasite fauna [54].

In Africa, there are more than 100 species of *Dactylogyrus* recorded from cyprinoid fishes with 15 descriptions from South African cyprinoids [1, 37, 53, 55]. To date, only six species have been described and/or reported from *E. paludinosus. Dactylogyrus afrosclerovaginus* Paperna, 1973, described from *Enteromius neglectus* (Boulenger, 1903) in Uganda, and also reported from *E. paludinosus* in South Africa [36]. *Dactylogyrus afrochelatus* Paperna, 1973 described from *Enteromius amphigramma* (Peters, 1852) (junior synonym of *E. paludinosus*) in Kenya but not recorded in South Africa. *Dactylogyrus afropsilovaginus* Paperna, 1973 and *Dactylogyrus clavatovaginus* Paperna, 1973 both described from *E. paludinosus* in Uganda [47, 48], neither of which has been reported in South Africa*. Dactylogyrus dominici* Mashego, 1983 and *Dactylogyrus teresae* Mashego, 1983 were described from *E. paludinosus* in South Africa [36]*.* Concerning molecular data, only 25 *Dactylogyrus* spp. (~25%) collected from Africa have sequence data available on GenBank. None of the *Dactylogyrus* species recorded from *E. paludinosus* have been genetically characterised, and from South Africa, only *Dactylogyrus matlopong* Acosta, Truter, Malherbe & Smit, 2022 occurring on *Labeobarbus aeneus* (Burchell, 1822), has representative sequence data [1].

Dactylogyrids identified as *D. dominici* and *D. teresae* were recently collected from *E. paludinosus* in the Vaal River system, Gauteng, South Africa. Both of these species were described from *E. paludinosus* in the Turfloop and Seshego dams in Limpopo, South Africa respectively [36] and later reported in the Barberspan Wetland [70] and Middle Letaba Dam [45]. The descriptions were based on standard light microscopy with limited point-to-point measurements. Moreover, the three-dimensional morphology of the sclerotised structures of these helminths has not been studied using SEM, and there are no genetic data available for either species. This study was, therefore, conducted to update the taxonomic information for *D. dominici* and *D. teresae* using light microscopy (LM) for comparison with previous studies and type material, coupled with SEM of taxonomically important isolated sclerotised structures, and finally genetic characterisation of both species for the first time*.* Furthermore, for the first time, sclerotised structures of GAP-mounted material were isolated and studied using SEM.

## Materials and methods

### Ethics

Fish were captured according to a permit granted by the Gauteng Department of Agriculture & Rural Development (permit number: CPE2 0118). They were euthanised by severing the spinal cord and double pithing according to the South African National Standard: Care and Use of Animals for Scientific Purposes [67] as approved by the University of Johannesburg Ethics Committee (ethics clearance reference number: 2022-02-02/Maduenyane_Oldewage).

### Sample collection and specimen preparation

Forty-one specimens of *E. paludinosus* (1.28–5.50 g) were collected from the Vaal River system, below the dam wall (26°52′12.38″ S; 28° 7′13.99″ E) using an electroshocker and hand nets, following the conditions of the permit above. The skin and fins of collected fish specimens were examined for helminths using a Zeiss Stemi 350 stereomicroscope (Carl Zeiss, Jena, Germany). Thereafter, fish were euthanised as detailed above. The gills were removed by dissection and examined for parasites, which were dislodged from the gill filaments using a dissection needle and collected with a micropipette. Isolated parasites were then mounted onto glass microscope slides with glycerine ammonium picrate (GAP) [35] and studied using LM. For SEM and molecular analysis, whole worms were stored in 96% ethanol (Sigma Aldrich, Darmstadt, Germany) and further processed as described below.

### Light microscopy of whole worms

**Figure 1:**
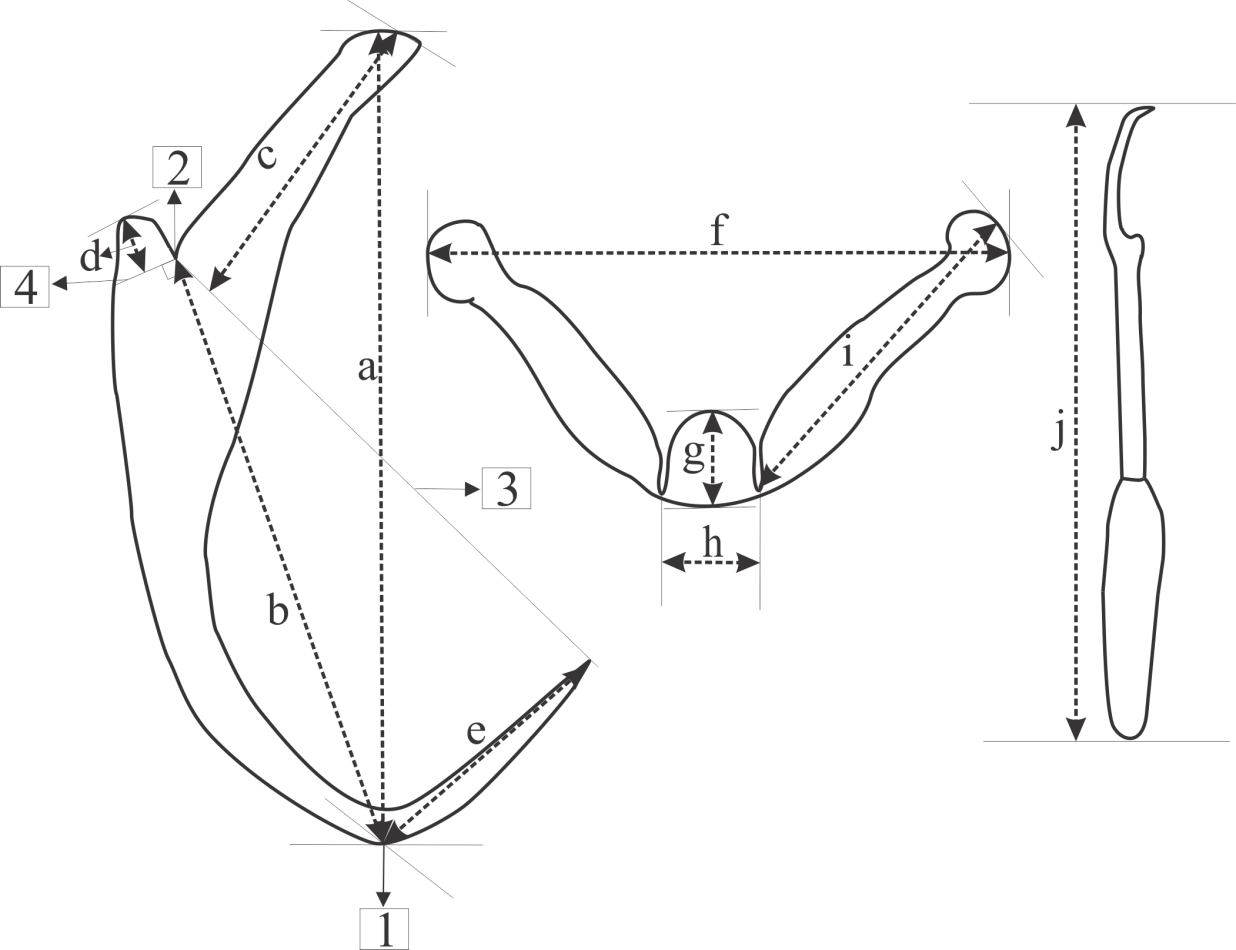
Point-to-point measurements conducted in the present study (illustrations not drawn to scale), adapted from Paperna, 1959. (a) – anchor total length; (b) – anchor shaft length; (c) – anchor inner root length; (d) – anchor outer root length; (e) – anchor point length; (f) – transverse bar total width; (g) – transverse bar total length; (h) – medial part length; (i) – transverse bar lateral arm length; (j) – hook total length; (1) – nadir of the anchor curve (2) – deepest point of the notch separating the anchor roots (3) – line connecting 2 and the tip of the anchor point (4) – line from 2 at 90 degrees to b.

**Figure 2:**
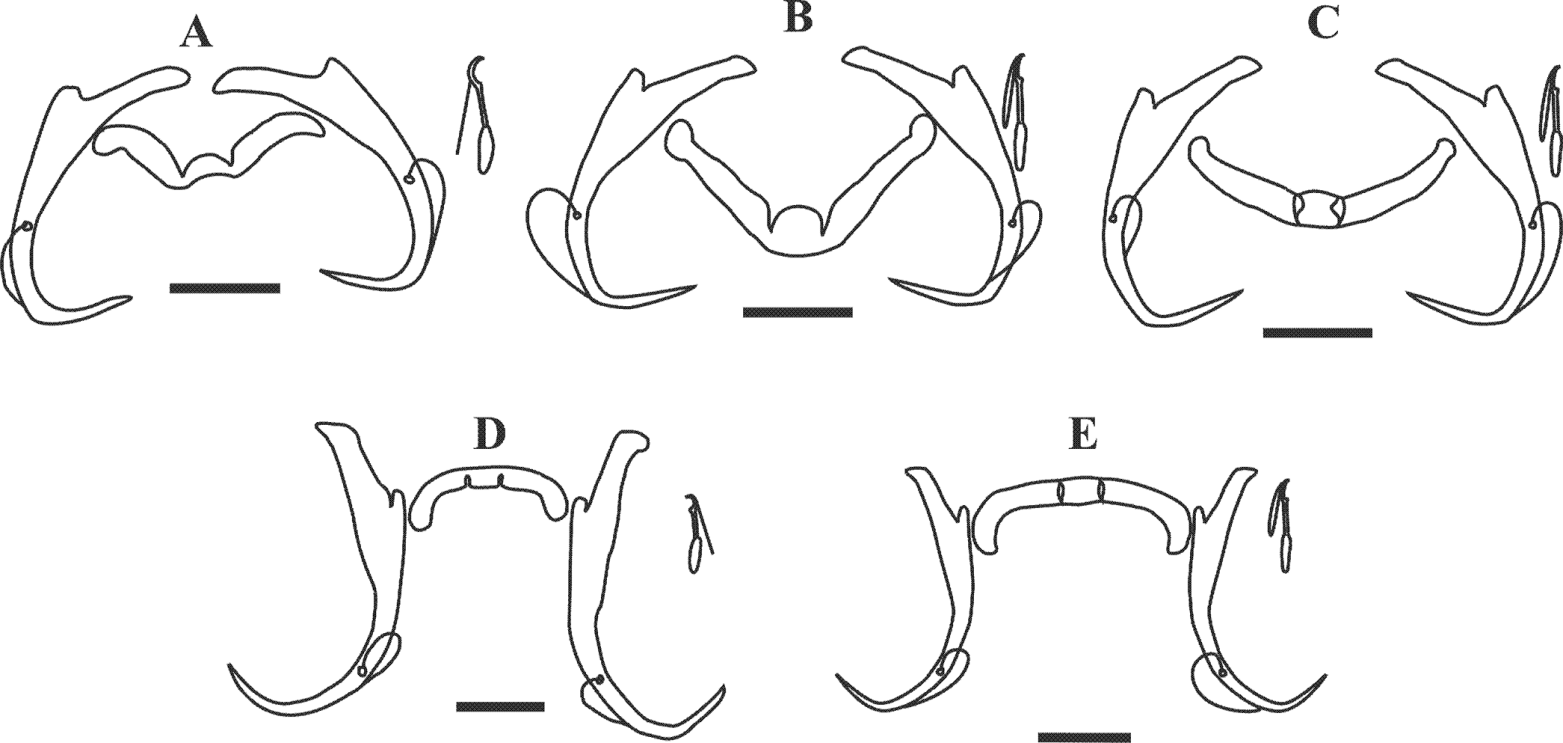
Line drawings of haptoral sclerites of *Dactylogyrus dominici* and *Dactylogyrus teresae* redrawn from Mashego (1983) and the present study (all 20 μm). (A) – *D. dominici* from Mashego (1983); (B) – *D. dominici* orientation 1 from the current study; (C) – *D. dominici* orientation 2 from the current study; (D) – *D. teresae* from Mashego (1983); (E) – *D. teresae* from the current study.

GAP-mounted material was examined using a Zeiss Axioplan 2 imaging light microscope with Axiovision 4.7.2 software. The point-to-point measurements of sclerotised structures were adapted from the drawings of Paperna [47] as follows (Fig. 1): (a) anchor total length (ATL) as the furthest distance between the nadir of the anchor curve (**1**) and anchor inner root; (b) anchor shaft length (ASL) as the distance between **1** and the deepest point of the notch separating the anchor roots (**2**); (c) anchor inner root length (AIRL) as the furthest distance between the anchor inner root perpendicular to **3** (the line connecting **2** and the tip of the anchor point); (d) anchor outer root length (AORL) as the furthest distance from the anchor outer root perpendicular to **4** (the line from **2** at 90 degrees to b); (e) anchor point length (APL) as the distance from **1** and the tip of the anchor point; (f) transverse bar total width (TBTW) as the furthest distance between the distal points of the transverse bar; (g) transverse bar total length (TBTL) as the widest distance at the medial part of the transverse bar; (h) medial part length (MPL) as the distance between the two submedian folds of the transverse bar; (i) transverse bar lateral arm length (LAL) as the distance between the distal end of the lateral arm and the deepest point of the submedian fold; (j) hook total length (HTL) as the distance between the distal end of the hook shaft and the distal end of the hook sickle. Hooks were numbered following Mizelle & Klucka [38]. Obtained z-stacked light micrographs were uploaded onto CorelDraw 6 [69] and line drawings of the sclerotised structures were created by tracing the outlines of the images. Generated line drawings and measurements (presented as mean ± standard deviation (minimum–maximum)) were compared to measurements presented in the original description [36] (see Fig. 2 and Table 1). Additionally, the holotype and paratype specimens deposited in the Ditsong National Museum of Natural History (TM12372 A&B; TM12374 A&B) were obtained and studied as described above. Data from the type series and the present material were statistically compared using IBM SPSS version 29. The data were tested for normality using the Shapiro–Wilk test and histograms. The latter showed the data to be parametric; thus, an independent samples *t*-test was done.

**Table 1:** Measurements of *Dactylogyrus dominici* Mashego, 1983 and *Dactylogyrus teresae* Mashego, 1983 from the present study based on LM and SEM micrographs, compared with those presented in the descriptions by Mashego (1983) and measurements of the type series (TM12372 A&B; TM12374 A&B) studied here.

Reference	*D. dominici* Mashego, 1983				*D. teresae* Mashego, 1983			
	Present study SEM	Present study LM	Mashego [37]	Type material	Present study SEM	Present study LM	Mashego [37]	Type material
Body								
Total Length	–	413 ± 89 (205–586)	218–419	360 ± 28 (316–389)	–	405 ± 83.3 (231–487)	238–413	336 ± 25 (317–376)
Total Width	–	73 ± 26 (28–142)	31–75	63 ± 4 (59–70)	–	72 ± 19 (45–100)	38–69	78 ± 7 (69–89)
Anchors								
Total length	50 ± 1 (48–51)	51 ± 1 (48–55)	58–80	54 ± 6 (49–64)	73 ± 0.3 (72–73)	74 ± 2 (71–77)	100–106	72 ± 1 (70–74)
Shaft length	39 ± 0.3 (38–39)	40 ± 1 (37–42)	40–54	38 ± 0.7 (37–39)	60 ± 1 (59–62)	62 ± 2 (58–65)	69–75	59 ± 3 (55–61)
Inner root length	18 ± 0.4 (18–19)	19 ± 1 (18–23)	–	19 ± 0.8 (18–20)	20 ± 0.5 (19–20)	20 ± 0.8 (18–21)	23–29	21 ± 1 (19–23)
Outer root length	3 ± 0.9 (2–4)	4 ± 0.5 (3–6)	–	4 ± 0.6 (3–5)	5 ± 0.6 (4–5)	5 ± 0.4 (4–6)	6 –8	5 ± 1 (5–7)
Point length	17 ± 0.7 (16–17)	16 ± 0.7 (15–18)	15–19	16 ± 0.8 (15–17)	18 ± 1 (17–20)	20 ± 2 (17–22)	13–19	19 ± 0.7 (18–19)
Transverse bar								
Total width	43 ± 2 (41–44)	43 ± 6 (31–54)	43–58	41 ± 3 (38–43)	37 ± 1 (36–39)	41 ± 3 (36–46)	44–50	43 ± 3 (40–45.9)
Total length	5 ± 0.4 (5–6)	5 ± 1 (3–7)	4–5	5 ± 0.6 (5–6)	4 ± 0.6 (4–5)	5 ± 0.6 (4–6)	4–6	5 ± 0.3 (4–5)
LAL	22 ± 1 (20–23)	21 ± 2 (19–26)	–	21 ± 2 (19–23)	19 ± 0.9 (17–9)	21 ± 1.4 (19–22)	–	19 ± 1 (18–20)
MPL	10 ± 0.6 (9–10)	9 ± 0.7 (7–10)	–	9 ± 1 (8–10)	8 ± 0.7 (7–9)	8 ± 0.7 (7–9)	–	8 ± 0.4 (8–9)
Hooks						**7 pairs**	**8 pairs**	
Average	17–22	17–25	19–25	18–23	17–20	16–25	18–25	17–24
I	–	18 ± 0.6 (17–19)	–	–	–	17 ± 0.7 (16–19)	–	–
II	–	22 ± 0.6 (20–23)	–	–	–	22 ± 0.5 (21–23)	–	–
III	–	21 ± 0.7 (20–23)	–	–	–	21 ± 0.4 (21–22)	–	–
IV	–	20 ± 0.6 (19–21)	–	–	–	20 ± 0.3 (20–21)	–	–
V	–	18 ± 0.5 (17–19)	–	–	–	19 ± 0.4 (18–20)	–	–
VI	–	19 ± 0.4 (18–20)	–	–	–	20 ± 0.4 (19–20)	–	–
VII	–	23 ± 0.6 (22–25)	–	–	–	23 ± 1 (20–25)	–	–
Male copulatory organ								
Copulatory duct	46 ± 2 (42–47)	46 ± 3 (39–50)	25–45	46 ± 3 (42–48)	43 ± 0.2 (42–43)	43 ± 4 (38.4–52.2)	20 (28–31)	–
Accessory piece	26 ± 2 (23–27)	28 ± 1 (25–31)	15–19	28 ± 2 (26–31)	28 ± 0.6 (27–28)	27 ± 1 (26–30)	14–19	26 (n = 1)
Hook length	6 ± 0.4 (6–7)	7 ± 0.9 (5–8)	–	7 ± 0.6 (6–7)	8 ± 0.8 (8.3–8.4)	7 ± 1 (5–8)	–	7 (n = 1)
Vagina								
Total length	16 (n = 1)	12 ± 2 (7–18)	11–16	14 ± 0.5 (13–14)	19 ± 0.9 (18–19)	21 ± 3 (16–25)	28–31	20 ± 4 (15–24)
Total width	7 (n = 1)	6 ± 1 (5–10)	4–6	7 ± 0.6 (6–7)	18 ± 3 (15–20)	21 ± 4 (15–28)	13	20 ± 4 (17–27)

En-dash – measurements not done.

MPL: Medial part length; LRL: Lateral arm length.

### Scanning electron microscopy of isolated sclerotised structures

For the isolation of sclerotised structures, individual worms previously stored in 96% ethanol were placed on concavity slides with Tris EDTA buffer overnight. Thereafter, three changes of the buffer were done over one-hour intervals and soft tissue was digested using 0.5 μL of digestion buffer from an E.Z.N.A.^®^ Tissue DNA kit (Omega Bio-Tek Inc., Norcross, GA, USA) following Dos Santos & Avenant-Oldewage [16] and Maduenyane *et al.* [33]. After the digestion was complete and the digestion buffer thoroughly rinsed with distilled water, digested soft tissue was collected and stored in a freezer for molecular analysis, while isolated sclerotised structures were dried in a desiccator. Once dried, the structures were coated with gold using an Emscope SC500 sputter coater (Quorum Technologies, Lewes, UK) and micrographs taken at 6–10 kV acceleration voltage with a TESCAN Vega 3 LMH SEM (Brno, Czech Republic). Point-to-point measurements (in μm) were obtained as described above from scanning electron micrographs. These measurements were statistically compared to the measurements obtained with LM. The data were tested for normality as described above and found to be parametric; therefore, an independent samples *t*-test was done.

### Isolation of sclerotised structures from GAP-mounted material

After the examination of GAP-mounted specimens with LM, mounted whole specimens were removed from the slides. This was done by scraping the nail varnish off with a sharp needle to free the coverslip. Thereafter, the coverslip was carefully removed, the worm picked up using an eyelash brush (eyelash glued to a toothpick) and placed onto a concavity slide for soft tissue digestion, as described above. In cases where the worm was too brittle to pick up, soft tissue digestion was done on the original slide after removing the coverslip. Once the soft tissue was digested away, rinsing and drying of isolated sclerotised structures and processing for examination using SEM was done, as described above. Digested soft tissue was retained to attempt molecular analysis.

### Genetic characterisation

#### Extraction & amplification

Genomic DNA was extracted from 14 specimens using an E.Z.N.A.^®^ Tissue DNA kit. Six *D. dominici* (two whole worms and digested material from four specimens examined with SEM) and eight *D. teresae* (3 whole worms and digested material from five specimens examined with SEM) were used. Genomic DNA was also extracted from previously digested tissue from GAP-mounted material. A fragment of rDNA spanning partial 18S rDNA, complete internal transcribed spacer (ITS) 1 and partial 5.8S rDNA, a fragment of partial 28S rDNA, as well as the cytochrome c oxidase subunit 1 (CO1) mtDNA were amplified. Each PCR reaction included 5 μL of genomic DNA, 15 μL of Taq 2 × Master Mix (AMPLIQON, Denmark), 8.8 μL of molecular biology grade water (AccuGENE^®^, Lonza, Walkersville, MD, USA), and 0.6 μL of the forward and reverse primers, respectively.

For 18S-ITS1-5.8S rDNA, the primers S1 (5′–ATT CCG ATA ACG AAC GAG ACT–3′) [65] and IR8 (5′–GCT AGC TGC GTT CTT CAT CGA–3′) [63] were used. PCR conditions for this fragment of rDNA were 94 °C for 1 min, then 40 cycles of 94 °C for 1 min, annealing at 53 °C for 1 min, then 72 °C for 1 min and 30 s and final elongation at 72 °C for 10 min. For the 28S rDNA, U178 (5′–GCA CCC GCT GAA YTT AAG–3′) and L1642 (5′–CCA GCG CCA TCC ATT TTC A–3′) [32] were used. PCR conditions were set according to Acosta *et al.* [1]. For the CO1 mtDNA region, primers GYRO_COXF1 (5′–GCT TTT TAC KYT WGA TCA YAA GCG–3′) and GYRO_COXR1 (5′–ACA TAC CAA AAT AAT GAA TWG GWA AAA AAC–3′) were designed. Due to the long span of this primer set (1229 bp), limited success was achieved and internal forward primer LCO1P (5′–TTT TTT GGG CAT CCT GAG GTT TAT–3′) [31] reverse primer HCO2198 (5′–TAA ACT TCA GGG TGA CCA AAA AAT CA–3′) [21] were used to generate shorter reads which could be assembled. PCR conditions for COI mtDNA reactions were as follows: 5 min at 94 °C then 40 cycles of 94 °C for 45 s, annealing at 46 °C for 45 s then 72 °C for 90 s and final elongation at 72 °C for 10 min. Following PCR, 1% agarose gel infused with SafeView^TM^ Classic (Applied Biological Materials Inc., Richmond, BC, Canada) was used to verify successful amplification and viewed with a SmartDoc^TM^ 2.0 gel visualisation and smartphone imaging system (Accuris Instruments, Edison, NJ, USA).

#### Sequencing and phylogeny

Amplification of the rDNA and mtDNA from specimens prepared in GAP was unsuccessful. For sequencing of amplicons from the rest of the material, standard BigDye chemistry was used with an ABI 3137 Automated Sequencer (Applied Biosystems, Foster City, CA, USA). Geneious Prime version 2022.2.2 [29], MAFFT [28] and MEGA 7 and 11 [30, 68] were used to check, edit, if necessary, align and assemble obtained sequences. The sequences generated using the COI mtDNA primers (GYRO_COXF1 with HCO2198 and LCO1P with GYRO_COXR1) did not overlap, therefore N-nucleotides were used to fill the gaps in correlation to reference sequences using the full span of the designed primers. Phylogenetic analyses were performed using only the 18S-ITS1-5.8S rDNA and the 28S rDNA as there was limited and mostly unpublished sequence data for CO1 mtDNA available. Ten *Dactylogyrus* species closest to the generated data were selected using the Basic Local Alignment Search Tool (BLAST) [2] for each marker (Table 2). Additionally, sequence data of all *Dactylogyrus* species collected in Africa was included. Only sequences from peer-reviewed publications and those that covered 80% of the respective alignments were included in the analyses. *Diplectanum aequans* (Wagener, 1857) was used as an outgroup. Genetic distances based on the number of base pair (bp) differences and uncorrected *p*-distances were computed in MEGA 11. To reconstruct the evolutionary history, Bayesian inference (BI) was employed. The General Time Reversible model [41] was selected using the Model Finder [27] in IQ-Tree [42]. The data were analysed with 10 million Markov chain Monte Carlo generations using BEAST v2.5.0 [11], and 50% burn-in applied using Tree Annotator v2.5.0. The resultant topology was visualised using FigTree v1.4.3 and posterior probabilities were indicated at the respective nodes. Maximum likelihood analyses were also performed, but the topologies were uninformative due to low nodal support. Generated sequences for *D. dominici* and *D. teresae* were submitted to GenBank (18S-ITS1-5.8S: PQ834535–PQ834536; 28S: PQ834491–PQ834495; CO1: PQ845134–PQ845140).

**Table 2:** List of *Dactylogyrus* species used in phylogenetic analyses, their cyprinid hosts, locality (country), GenBank accession numbers and references.

*Dactylogyrus* species	Host	Host family	Specimen locality	Accession	Reference
*D. alatus* Linstow, 1878	*Telestes muticellus* (Bonaparte, 1837)	Leuciscidae	Italy	MK434946 *	[5]
	*Alburnus neretva* Buj, Šanda & Perea, 2010		Bosnia and Herzegovina	MG792956 *	[6]
				MG792957 *	[6]
*D. anchoratus* (Dujardin, 1845)	*Cyprinus carpio* Linnaeus, 1758	Cyprinidae	Czech Republic	AJ490161 **	[63]
	*Carassius auratus* (Linnaeus, 1758)			AJ564111 **	[61]
			Croatia	KY859795 **	[8]
*D. aspili* Birgi & Lambert, 1987	*Enteromius macrops* (Boulenger, 1911)		Senegal	KY629359 *	[59]
*D. atlasensis* El Gharbi, Birgi & Lambert, 1994	*Luciobarbus pallaryi* (Pellegrin, 1919)		Morocco	KY629337 **	[59]
				KY629356 **	[59]
*D. balkanicus* Dupont & Lambert, 1986	*Barbus tyberinus* Bonaparte, 1839		Italy	MN974248 **	[10]
	*Barbus plebejus* Bonaparte, 1839		Croatia	MG792861 **	[6]
	*Barbus rebeli* Koller, 1926		Albania	MG792863 **	[6]
	*Barbus prespensis* Karaman, 1924			KY201093 **	[8]
*D. benhoussai* Rahmouni, Řehulková & Šimková, 2017	*Luciobarbus moulouyensis* (Pellegrin,1924)		Morocco	KX553862 **	[52]
				KX578025 **	[52]
*D. borealis* Nybelin, 1937	*Phoxinus bigerri* Kottelat, 2007		Spain	MN338222 *	[4]
	*Phoxinus* sp.		Bosnia and Herzegovina.	KY629372 *	[59]
*D. borjensis* El Gharbi, Birgi & Lambert, 1994	*Luciobarbus zayanensis* Doadrio, Casal-López & Yahyaoui, 2016		Morocco	MN973819 **	[10]
				MN974257 **	[10]
*D. brevicirrus* Paperna, 1973	*Labeo parvus* Boulenger, 1902		Senegal	KY629362 **	[59]
*D. cf. parvicirrus* Seamster, 1948	*Notemigonus crysoleucas* (Mitchill, 1814)	Leuciscidae	New York, USA	OM108563 **	[64]
*D. cheloideus* Rogers, 1967	*Rhinichthys atratulus* (Hermann, 1804)		Wisconsin, USA	OM108567 **	[64]
*D. crivellius* Dupont & Lambert, 1986	*Barbus tyberinus* Bonaparte, 1839	Cyprinidae	Italy	MK434929 **	[5]
	*Barbus plebejus* Bonaparte, 1839		Croatia	MG792862 **	[6]
	*Barbus* sp.		Albania	MG792866 **	[6]
	*Barbus rebeli* Koller, 1926			MG792864 **	[6]
	*Barbus peloponnesius* Valenciennes, 1842		Greece	KY629339 **	[59]
	*Barbus prespensis* Karaman, 1924		Albania	KY201094 **	[8]
	*Barbus balcanicus* Kotlík, Tsigenopoulos, Ráb & Berrebi, 2002		Greece	MG792854 **	[6]
			Bulgaria	EF582617 **	[62]
***D. dominici* Mashego, 1983**	***Enteromius paludinosus* (Peters, 1852)**		**South Africa**	PQ834535 *	**Present study**
				PQ834491 **	
				PQ834492 **	
*D. draaensis* El Gharbi, Birgi & Lambert, 1994	*Luciobarbus lepineyi* (Pellegrin, 1919)		Morocco	MN974258 **	[10]
				MN973816 **	[10]
*D. extensus* Mueller & Van Cleave, 1932	*Cyprinus carpio* Linnaeus, 1758		China	AY553629 **	[71]
			Japan	LC764381 **	[43]
			Egypt	MZ352176 **	[3]
			Czech Republic	AJ969944 **	[60]
			Iran	MF288785 **	[15]
			Czech Republic	AJ564129 **	[59]
*D. falciformis* Achmerov, 1952	*Cyprinus carpio* Linnaeus, 1758		Czech Republic	MZ031061 *	[9]
				MZ031072 **	[9]
*D. falcilocus* Guégan, Lambert & Euzet, 1988	*Labeo coubi* Rüppell,1832		Senegal	KY629365 **	[59]
*D. falsiphallus* Rahmouni, Řehulková & Šimková, 2017	*Luciobarbus maghrebensis* Doadrio, Perea & Yahyaoui, 2015		Morocco	KX553861 **	[52]
				KX578024 **	[52]
*D. fallax* Wagener, 1857	*Leuciscus cephalus* (Linnaeus, 1758)	Leuciscidae	Czech Republic	AJ564132 **	[61]
	*Rutilus rutilus* (Linnaeus, 1758)			AJ564131 **	[61]
				MG792906 **	[6]
	*Chondrostoma nasus* (Linnaeus, 1758)			MG792872 **	[6]
	*Vimba vimba* (Linnaeus, 1758)			KY629341 **	[59]
*D. fimbriphallus* El Gharbi, Birgi & Lambert, 1994	*Luciobarbus massaensis* Pellegrin, 1922	Cyprinidae	Morocco	KY629332 **	[59]
				KY629357 **	[59]
*D. flagristylus* Chien, 1974	*Nocomis biguttatus* (Kirtland, 1840)	Leuciscidae	Wisconsin, USA	OM108566 **	[64]
*D. inexpectatus* Izjumova, 1955	*Carassius auratus* (Linnaeus, 1758)	Cyprinidae	Czech Republic	AJ969945 *	[60]
*D. ksibii* El Gharbi, Birgi & Lambert, 1994	*Luciobarbus ksibi* Boulenger, 1905		Morocco	MN973812 **	[10]
				MN974252 **	[10]
				MN973817 **	[10]
				KX553864 **	[52]
				MN973811 **	[10]
				KX578027 **	[52]
				MN974251 **	[10]
				MN974250 **	[10]
*D. kulindrii* El Gharbi, Birgi & Lambert, 1994	*Carasobarbus fritchii* (Günther, 1874)		Senegal	KY629336 **	[59]
				KY629354 **	[59]
*D. leonis* Musilová, Řehulková & Gelnar, 2009	*Labeo coubi* Rüppell,1832		Senegal	KY629360 **	[59]
*D. marocanus* El Gharbi, Birgi & Lambert, 1994	*Carasobarbus fritchii* (Günther, 1874)		Morocco	KY629355 **	[59]
				KY629333 **	[59]
	*Luciobarbus zayanensis* Doadrio, Casal-López & Yahyaoui, 2016			MW218669 **	[54]
				MW218671 **	[54]
	*Pterocapoeta maroccana* Günther, 1874			MW218579 **	[54]
				MW218672 **	[54]
	*Luciobarbus ksibi* Boulenger, 1905			MW218580 **	[54]
				MW218673 **	[54]
*D. matlopong* Acosta, Truter, Malherbe & Smit, 2022	*Labeobarbus aeneus* (Burchell, 1822)		South Africa	ON391043 *	[1]
				ON391042 **	[1]
*D. minutus* Kulwiec, 1927	*Cyprinus carpio* Linnaeus, 1758		Iran	MF926269 *	[15]
*D. oligospirophallus* Paperna, 1973	*Labeo coubi* Rüppell,1832		Senegal	KY629361 **	[59]
*D. oryziasi* Nitta & Nagasawa, 2017	*Oryzias latipes* (Temminck & Schlegel, 1846)	Adrianichthyidae	Japan	LC190737 *	[44]
*D. propinquus* Bychowsky, 1931	*Abramis sapa* (Pallas, 1814)	Leuciscidae	Czech Republic	AJ564147 **	[61]
*D. ramulosus* Malevitskaia, 1941	*Aspius aspius* (Linnaeus, 1758)		Czech Republic	AJ564149 **	[61]
*D. rarissimus* Gusev, 1966	*Alburnus neretvae* Buj, Šanda & Perea, 2010		Bosnia and Herzegovina	MG792958 **	[6]
				MG792959 **	[6]
	*Rutilus lacustris* (Bonaparte, 1841)		Greece	MG793015 **	[6]
	*Alburnus arborella* (Bonaparte, 1841)		Italy	MK434947 **	[5]
	*Telestes fontinalis* (Karaman, 1972)		Croatia	MG792997 **	[6]
	*Telestes dabar* Bogutskaya*,* Zupančič, Bogut & Naseka, 2012		Bosnia and Herzegovina	MG793056 **	[6]
	*Rutilus basak* (Heckel, 1843)			MG793011 **	[6]
	*Telestes metohiensis* (Steindachner, 1901)			MG793059 **	[6]
	*Sarmarutilus rubilio* (Bonaparte, 1837)		Italy	MK455795 **	[5]
	*Pelasgus laconicus* (Kottelat & Barbieri, 2004)		Greece	MG793006 **	[6]
	*Rutilus ohridanus* (Karaman, 1924)		Albania	MG793019 **	[6]
*D. senegalensis* Paperna, 1969	*Labeo senegalensis* Valenciennes, 1842	Cyprinidae	Senegal	KY629363 **	[59]
*D. scorpius* Rahmouni, Řehulková & Šimková, 2017	*Luciobarbus rifensis* Doadrio, Casal-López & Yahyaoui, 2015		Morocco	KX553860 **	[52]
				KX578023 **	[52]
*D. soufii* (Lambert, 1977)	*Telestes montenigrinus* (Vukovic, 1963)	Leuciscidae	Albania	MG792946 **	[6]
*Dactylogyrus* sp*.*	*Enteromius niokoloensis* (Daget, 1959)	Cyprinidae	Senegal	KY629358 **	[59]
***D. teresae* Mashego. 1983**	***Enteromius paludinosus* (Peters, 1852)**		**South Africa**	PQ834536 **	**Present study**
				PQ834493 *	
				PQ834494 *	
				PQ834495 *	
*D. titus* Guégan, Lambert & Euzet, 1988	*Labeo senegalensis* Valenciennes, 1842		Senegal	KY629364 **	[59]
*D. varius* Rahmouni, Řehulková & Šimková, 2017	*Luciobarbus maghrebensis* Doadrio, Perea & Yahyaoui, 2015		Morocco	KX553863 **	[52]
				KX578026 **	[52]
	*Squalius lucumonis* (Bianco, 1983)	Leuciscidae	Italy	MK434959 **	[5]
*D. vastator* Nybelin, 1924	*Carassius gibelio* (Bloch, 1782)	Cyprinidae	Czech Republic	KY629366 *	[59]
			Croatia	MZ031059 *	[9]
				MW443031 *	[7]
	*Cyprinus carpio* Linnaeus, 1758		Iran	MF928712 *	[15]
	*Barbus plebejus* Bonaparte, 1839		Italy	MK434948 *	[5]
	*Aulopyge huegelii* Heckel, 1843		Bosnia and Herzegovina	KY201106 *	[8]
*D. volutus* El Gharbi, Birgi & Lambert, 1994	*Carasobarbus fritchii* (Günther, 1874)		Morocco	KY629353 **	[59]
				KY629334 **	[59]
*D. yinwenyingae* Gusev, 1962	*Squalius lucumonis* (Bianco, 1983)	Leuciscidae	Italy	MK434959 **	[5]
*D. zatensis* El Gharbi, Birgi & Lambert, 1994	*Carasobarbus fritchii* (Günther, 1874)	Cyprinidae	Morocco	KY629352 **	[59]
				KY629335 **	[59]

* 28S rDNA sequences.

** 18S-ITS1-5,8S rDNA sequences.

## Results

### Morphometry

All specimens examined in the present study conformed to the diagnostic criteria for *Dactylogyrus* by Bykhovskaya-Pavlovskaya *et al.* [13]. The results obtained from LM confirmed the presence of two species of *Dactylogyrus* on the gills of *E. paludinosus* in the Vaal River, identified as *D. dominici* and *D. teresae* due to their similarity to the descriptions by Mashego [36]. Both dactylogyrids have elongated bodies with a thin and translucent tegument divided into a body trunk, a peduncle and a haptor. The anterior of the trunk bears two pairs of bilateral lobes and two pairs of eyespots. The adult worms have a sclerotised male copulatory organ (MCO) posterior to the pharynx, which comprises a coiled copulatory duct adjoined to a bulbous base of the accessory piece. From the bulbous base of the MCO, the copulatory duct proceeds to the proximal end. Posterior to the MCO is a sclerotised vagina that lies adjacent to the dextral side of the body wall. The haptor is situated at the posterior end, and bears three types of sclerites: a pair of anchors, a transverse bar, and seven pairs of hooks of varying sizes.

Measurements obtained from flattened specimens with LM and isolated sclerotised structures with SEM were highly similar for both species, respectively. However, significant differences for the respective hook total lengths could not be calculated as hook numbering could not be accurately attributed post-digestion, due to the shifting of sclerite positions. Similarly, the point-to-point measurements of sclerotised structures obtained using LM for both the study material and type specimens of both respective species in this study were highly similar. All *p*-values indicated non-significance (*p* ˃ 0.05) except for the anchor total length of *D. dominici* between type specimens and study material (*p* < 0.001). The significance of the isolated vaginal length and width of *D. dominici* could not be compared to LM data as only one specimen was measured post-digestion. Respective *p*-values are presented in Supplementary Table S1.

Type specimens were deteriorated; hence, some measurements could not be obtained. The holotype specimen for *D. dominici* was dried out and the copulatory organs could not be seen. However, the copulatory organs were examined in some of the paratype specimens and measurements were obtained. Moreover, only two hooks could be measured for the holotype, with all paratypes having a limited number of hooks visible. For the holotype of *D. teresae,* most of the measurements were obtained except for measurements of the MCO as it was not visible. The MCO was visible in only one paratype specimen, but the copulatory duct (cirrus axis) was not visible and thus not measured. Only four hooks could be measured for the holotype specimen, and no paratypes had a complete set of hooks visible. For both species, the designations of the hooks could not be determined and were also lacking from the original descriptions. Thus, the hook measurements of the type material were not comparable to the study material.

## *Dactylogyrus dominici* Mashego, 1983

Synonyms: *Dactylogyrus* sp. 1 [70]; *Dactylogyrus* sp. 3 [70]

Type host: *Enteromius paludinosus* (Peters, 1852) Type locality: Turfloop Dam, Limpopo, South Africa [36]

Other localities: Barberspan Wetland, North West, South Africa [70]; Middle Letaba Dam, Limpopo, South Africa [36]

New locality: Vaal River, Gauteng, South Africa (26° 52′12.38″ S; 28° 7′13.99″ E)

Infection site: Gills

Material deposited: Ten GAP mounted voucher specimens were deposited to the Ditsong National Museum of Natural History, Pretoria (Gen Ent 0346-51 & Gen Ent 0353-54), to the Iziko Museums of South Africa, Cape Town (SAMC-A097106-110 & SAMC-A097112-113) and to the Natural History Museum, London (NHMUK2025.1.3.1-8 & NHMUK2025.1.3.10-11), respectively.

Sequence data: 18S-ITS1-5.8 rDNA: PQ834535; 28S rDNA: PQ834491–PQ834492; CO1 mtDNA PQ845134–PQ845135.

**Figure 3:**
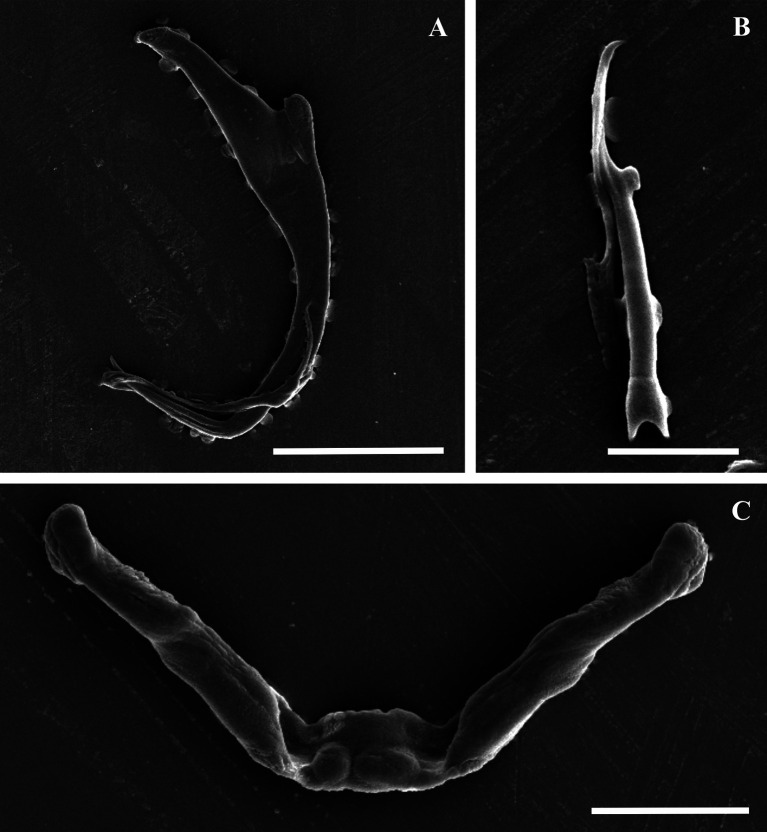
Scanning electron micrographs of the isolated haptoral sclerites of *Dactylogyrus dominici* Mashego, 1983*.* (A) – anchor (20 μm; from ethanol fixed specimen); (B) – hook (5 μm; from ethanol fixed specimen); (C) – transverse bar (10 μm; from previously mounted GAP specimen).

**Figure 4:**
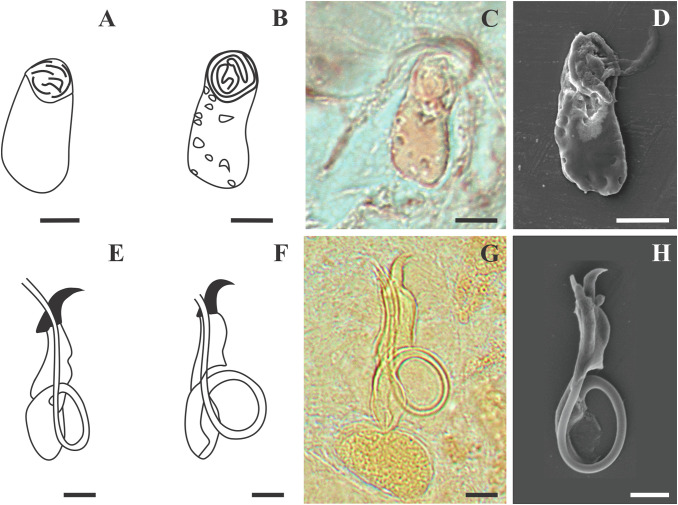
Line drawings, light and scanning electron micrographs of the male copulatory organ and vagina of *Dactylogyrus dominici* Mashego, 1983 from this study compared with line drawings from the original description. (A & E) – line drawings of vagina and MCO redrawn from Mashego (1983); (B & F) – line drawings of vagina and MCO from present study; (C & G) – light micrograph of vagina and MCO from present study; (D & H) – scanning electron micrograph of vagina and MCO (dorsal view) from previously mounted GAP specimens in present study (scale value for all drawings and micrographs of vagina 5 μm and MCO 10 μm).

Morphology based on 37 flattened whole worms mounted in GAP: Total body 413 ± 89 (206–586) long, 73 ± 26 (28–142) wide. Haptor with pair of anchors 51 ± 1 (48–55) long (Figs. 2A–2C and 3A), shaft length 40 ± 1 (37–42), inner root length 19 ± 1 (18–23), outer root length 4 ± 0.5 (3–6), point length 16 ± 0.7 (15–18). Anchors connected by transverse bar with two orientations 43 ± 6 (31–54) long, 5 ± 0.9 (3–7) wide (Figs. 2A–2C and 3C). First orientation (Fig. 2B) identical to the drawing presented in Mashego ([36]; Fig. 2A), bar comprises two sub-median folds dividing it into a medial part conjoined to upward-facing, lateral arms with rounded knob-like ends. Second orientation (Fig. 2C), with lateral arms pointing at an angle to the medial part and also lacks rounded knob-like ends. Both orientations measured 9 ± 0.7 (7–10) long, medial part, 22 ± 2 (19–26) long flexible lateral arms. Seven pairs of hooks (Figs. 2A–2C and 3B) varying in total length (17–25), with sclerotised blade, short fibrous handle and sclerotised shaft connected to semi-sclerotised wider base. First pair 18 ± 0.6 (17–19) long, second pair 22 ± 0.6 (20–23) long, third pair 21 ± 0.7 (20–23) long, fourth pair 20 ± 0.6 (17–21) long, fifth pair 18 ± 0.5 (17–19) long, sixth pair 19 ± 0.4 (18–20) long, seventh pair 23 ± 0.6 (22–25) long. No needles (4A hooks) were observed. Vagina (Figs. 4A–4D) 12 ± 2 (7–18) long, 6 ± 1 (5–10) wide, almost reniform, consisting of two unequal parts, the larger superficially dented and the smaller rounded. Vagina connected to a thinly lined pouch extending into body in some specimens. Accessory piece of MCO (Figs. 4E–4H) 28 ± 1 (25–31) long, terminal hook 7 ± 0.9 (5–8) long, copulatory duct (cirrus axis) 46 ± 3 (39–50) long.

### Remarks

The gross morphology of specimens of *D. dominici* from the current study are identical to that of the type material and the description by Mashego [36], except for the vagina. The vagina was not visible in the holotype, but was visible in three paratype specimens, with two specimens matching the line drawing presented in [36] and one differing in that it is more elongated with one end round and the other end pointed resembling the vagina of *Dactylogyrus clavatovaginus* Paperna, 1973 [48, 49]. The line drawing and measurements of the vagina presented by Mashego [36] matches that of specimens from this study. Additionally, most of the measurements in the original description, of the type material and the current study were mostly similar, with only a few discrepancies. The total and shaft lengths of the anchors presented by Mashego [36] were longer (total length: 48–55 in present study; 49–64 in type material; 58–80 in [36]. Shaft length: 37–42 in present study; 37–39 in type material; 40–54 in [36]). Hooks were not numbered or measured in pairs in the original description [36], only the range of the total length was given which overlapped with the measurements obtained here. The accessory piece of the MCO was larger in specimens from this study in comparison to the description (28 (25–31) in present study; 28 (26–31) in type material; 15–19 in [36]).

In the description of *D. dominici,* Mashego [36] remarks that this species resembles *D. afropsilovaginus.* However, *D. dominici* is more similar to *Dactylogyrus cf. clavatovaginus* reported by Paperna [48, 49] as the anchors, transverse bar, hooks, MCO and vagina are morphologically similar in that the anchors have long inner roots and short outer roots, the transverse bar in both species has two submedian folds, the hook has a slender shaft connected to a wider rounded base. The MCO comprises an accessory piece with a bulbous base connected to a copulatory duct that coils once and the anterior end of the MCO terminates with a hook. Lastly, the vagina consists of a dented reniform larger part and a smaller rounded part with hairlike projections. Most of the sclerite measurements do not correspond, only the anchor root measurements and the transverse bar width overlap. Whereas in *D*. *afropsilovaginus,* only the transverse bar is slightly similar to that of *D. dominici.* The morphological features of *D*. *dominici* are representative of the *Dactylogyrus afrobarbae* species group in agreement with Mashego [36].

## *Dactylogyrus teresae* Mashego, 1983

Type host: *Enteromius paludinosus* (Peters, 1852)

Type locality: Seshego Dam, Limpopo, South Africa [36]

Other localities: Barberspan Wetland, North West, South Africa [70]; Middle Letaba Dam, Limpopo, South Africa [36].

New locality: Vaal River, Gauteng, South Africa (26° 52′12.38″ S; 28° 7′13.99″ E)

Infection site: Gills

Material deposited: Three GAP mounted voucher specimens were deposited to the Ditsong National Museum of Natural History, Pretoria (Gen Ent 0353-54), two to the Iziko Museums of South Africa, Cape Town (SAMC-A097111), and two to the Natural History Museum, London (NHMUK2025.1.3.8-9), respectively.

Sequence data: 18S-ITS1-5.8 rDNA: PQ834536; 28S rDNA: PQ834493–PQ834495; CO1 mtDNA PQ845136–PQ845140.

**Figure 5:**
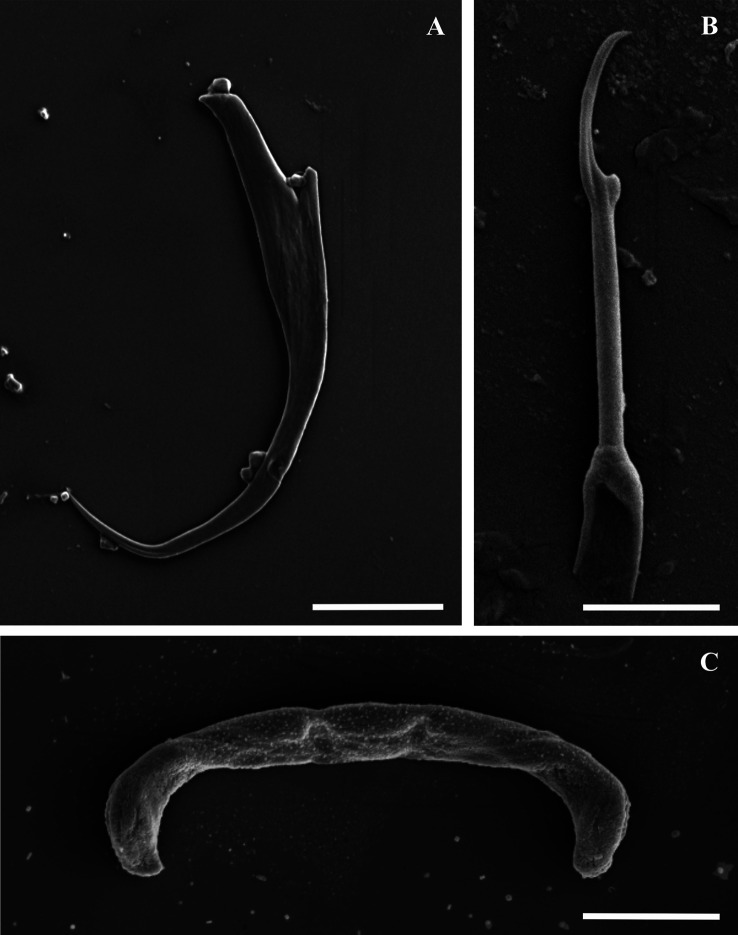
Scanning electron micrographs of the isolated haptoral sclerites of *Dactylogyrus teresae* Mashego, 1983*.* (A) – anchor (20 μm; from ethanol fixed specimen); (B) – hook (5 μm; from previously mounted GAP specimen); (C) – transverse bar (10 μm; from ethanol fixed specimen).

**Figure 6:**
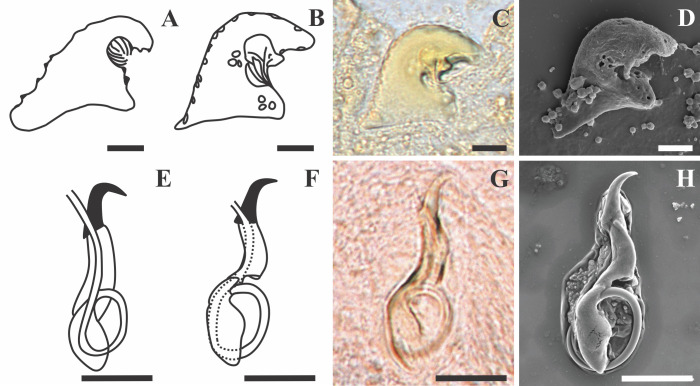
Line drawings, light and scanning electron micrographs of the male copulatory organ and vagina of *Dactylogyrus teresae* Mashego, 1983 from this study compared with line drawings from the original description. (A & E) – line drawings of vagina and MCO redrawn from Mashego (1983); (B & F) – line drawings of vagina and MCO from present study; (C & G) – light micrograph of vagina and MCO from present study; (D & H) – scanning electron micrograph of vagina and MCO (ventral view) from previously mounted GAP specimens in present study (scale value for all drawings and micrographs of vagina 5 μm and MCO 10 μm).

Morphology based on eleven flattened whole worms in GAP: Body 405 ± 83 (231–487) long, 72 ± 19 (45–100) wide. Haptor with pair of anchors 74 ± 2 (71–77) long (Figs. 2D, 2E and 5A), shaft length 62 ± 2 (58–65), inner root length 20 ± 0.8 (19–21), outer root length 5 ± 0.4 (4–6), point length 20 ± 2 (17–22). Anchors connected by elongated C-shaped transverse bar (Figs. 2D, 2E and 5C) 41 ± 3 (36–46) long, 5 ± 0.6 (4–6) wide with two sub-median folds dividing the bar into a medial part 8 ± 0.7 (7–9) long, connected to lateral arms 21 ± 1 (19–23) long with curved ends. Fourteen (seven pairs) hooks (Figs. 2D, 2E and 5B) of differing lengths comprising sclerotised blade with long fibrous handle, and sclerotised shaft connected to semi-sclerotised wider base. First pair 17 ± 0.7 (16–19), second pair 22 ± 0.5 (21–23), third pair 21 ± 0.4 (21–22), fourth pair 20 ± 0.3 (20–21), fifth pair 19 ± 0.4 (18–20), sixth pair 20 ± 0.4 (19–20), seventh pair 23 ± 1 (20–25). No needles (4A hooks) were observed. Sclerotised vagina (Figs. 6A–6D) 21 ± 3 (16–25) long, 21 ± 4 (15–28) wide, comprising two parts, the larger superficially dented, robustly falciform, the smaller with hair-like extensions on the concave margin. Accessory piece of MCO (Figs. 6E–6H) 27 ± 1 (26–30) long, terminal hook 7 ± 1 (5–8) long and copulatory duct 43 ± 4 (38–52) long.

### Remarks

Specimens of *D. teresae* from the present study match the morphology of the type material and the description by Mashego [36]. However, a few minor discrepancies in point-to-point measurements were observed. The anchor measurements corresponded with those of the type material studied here; however, these were smaller than those in [36] except for the anchor points, which ranged from 17 to 22 μm in specimens from the present study overlapping with those of the type material studied here 18–20 μm and those presented in Mashego [36] 13–19 μm. Regarding hooks, seven pairs were counted from specimens in the current study, whereas Mashego [36] counted eight. This could not be resolved with the type material as very few hooks were visible, however, the measurements of those hooks that were visible had similar variation in total lengths observed here for the different pairs. The copulatory duct was not measured in the type material as it could not be seen.

The original description mentions that *D. teresae* resembles *Dactylogyrus longionchus* Paperna, 1973. However, these two species are very distinct from each other, in that the anchors of *D. longionchus* have shorter inner roots and points. The transverse bar of *D. longionchus* is also very short and wide, lacking sub-median folds. The vagina is completely different from that of *D. teresae* as it is rounded, connected to an elongated leaf-like structure terminating in a pointed end [48, 49]. The only similarities between *D. dominici* and *D. longionchus* are the structure of the MCO and long anchor shafts [48, 49]. *Dactylogyrus teresae* has similar MCO and vaginal morphology to *Dactylogyrus allolongionchus* Paperna, 1973 but differs in anchor and transverse bar morphology, which is more similar between *D. teresae* and *D. afrochelatus* [48, 49]*.*

### *Dactylogyrus dominici* versus *Dactylogyrus teresae*

*Dactylogyrus dominici* and *D. teresae* share more morphological features with each other than with other dactylogyrids. These species have very similar anchors with long inner roots and very short outer roots. However, *D. teresae* has long and slightly curved anchor shafts, unlike the anchors of *D. dominici* which have relatively shorter shafts. The transverse bar of both *D. dominici* and *D. teresae* comprise two sub-medial folds; however, the medial part is flattened and wider in *D. teresae*, whereas it is raised in *D. dominici.* Moreover, in *D. teresae*, the bar is curved at the extreme ends, unlike in *D. dominici* where the bar is less curved and has lateral arms with knob-like ends. The fibrous handle of the hook is longer in *D. teresae* than in *D. dominici.* The hooks of *D. dominici* are robust with a slight bend, contrary to those of *D. teresae* which are slender, straight, and have a long blade. The MCO of both species is characteristic of the *D. afrobarbae* species group, that is, they both have a long, tubiform copulatory duct that coils once connected to an accessory piece which terminates with a hook. However, the MCO of *D. teresae* is smooth, without sharp edges on the accessory piece as seen in *D. dominici*, and the terminal hook is smooth and long. The vagina is the most distinct diagnostic feature between the species, as the vagina of *D. dominici* is reniform, but robustly falciform in *D. teresae*. Additionally, in *D. dominici*, the smaller part of the vagina (referred to as a prop in [36, 48, 49]) is terminal, whereas in *D. teresae* it is lateral on the concave margin.

### Genetic characterisation and phylogeny

**Figure 7:**
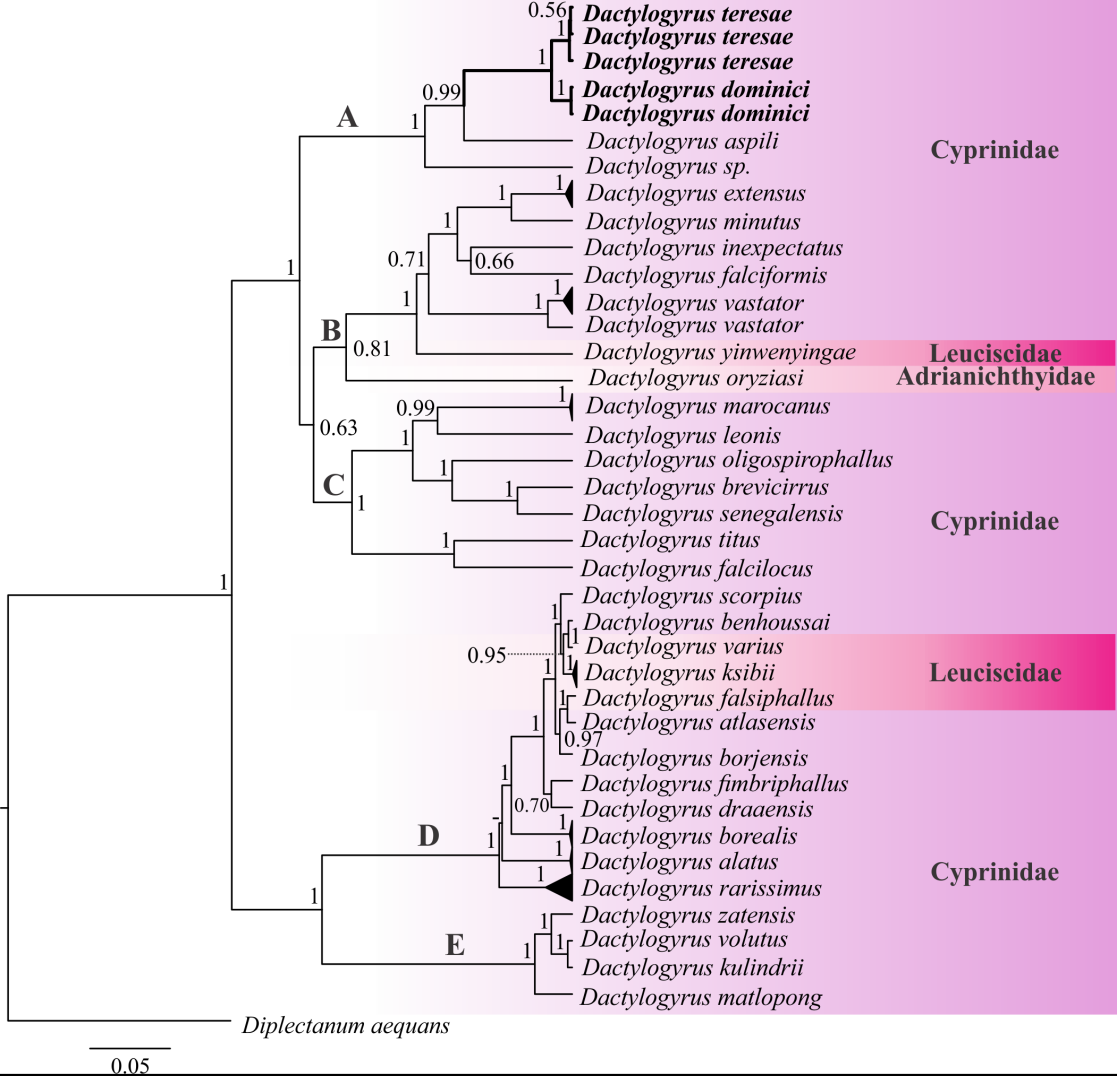
Bayesian inference (BI) phylogenetic topology inferred from partial 28S rDNA analysis of *Dactylogyrus* species. Posterior probabilities are indicated at branch nodes; nodes with less than 50% support are indicated with dashes. Data generated in the current study are in bold.

For 28S rDNA, two sequences from different specimens were obtained for *D. dominici* (1482–1508 bp) and three for *D. teresae* (1499–1508 bp). The alignment with selected *Dactylogyrus* sequences from GenBank was 815 bp long with 336 bp conserved, 425 bp variable, and 343 bp parsimony informative sites. Computed genetic variation (*p-*distances and bp differences) shown in Supplementary Table S2 between all distinct *Dactylogyrus* species included in the analyses (excluding data generated here) was 0.04–35.91%, whereas intraspecific distances of up to 2.7% were observed. There was no intraspecific variation between generated sequences for *D. dominici*, whereas there was 0–0.13% variability between sequences for *D. teresae* falling within the calculated intraspecific range. The genetic variation between *D. teresae* and *D. dominici* was 1.89–2.02%, falling within the interspecific range. The topology based on 28S rDNA (Fig. 7) show five major, well-supported clades. Clade A comprises four species including the data generated for *D. dominici* and *D. teresae,* all collected from *Enteromius* hosts. Clade B has seven species from *Cyprinus, Carassius, Barbus, Aulopyge, Squalius* and *Oryzias* hosts. Clade C has seven dactylogyrid species from *Labeo*, *Luciobarbus, Carasobarbus* and *Pterocapoeta* hosts. Clade D consists of twelve species, eight species from *Luciobarbus* and the rest from *Carassius, Phoxinus, Alburnus* and *Telestes* hosts. Clade E has four species collected from *Labeobarbus* and *Carasobarbus* hosts. All included taxa were collected from cyprinids and leuciscids except for one species, *Dactylogyrus oryziasi* Nitta & Nagasawa, 2017 which infects *Oryzias latipes* (Temminck & Schlegel, 1846) of the Adrianichthyidae Weber, 1913. *Dactylogyrus dominici* and *D. teresae* form a well-supported monophyletic group within clade A. Furthermore, these taxa grouped in a well-supported sister group to *D. aspili* Birgi & Lambert, 1987 (0.99 posterior probability) collected from *Enteromius macrops* (Boulenger, 1911), with *Dactylogyrus* sp*.* (KY629358) (1.0 posterior probability) from *Enteromius niokoloensis* (Daget, 1959) basal to clade A*.*

**Figure 8:**
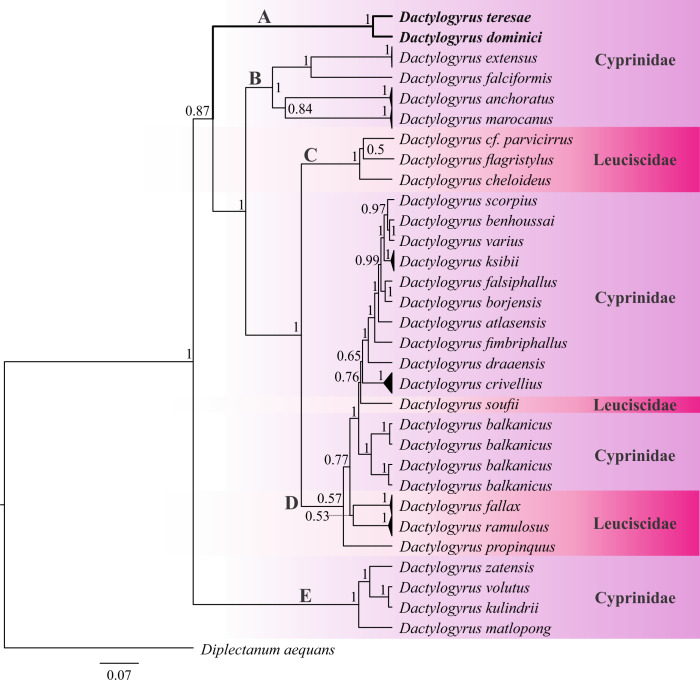
Bayesian inference (BI) phylogenetic topology inferred from partial 18S-ITS1-5.8S rDNA analysis of *Dactylogyrus* species. Posterior probabilities are indicated at branch nodes. Data generated in the current study are in bold.

For 18S-ITS1-5.8S rDNA, single sequences were obtained for *D. dominici* (920 bp) and *D. teresae* (918 bp) respectively. The alignment with selected *Dactylogyrus* sequences was 1433 bp long, with 609 bp conserved, 707 bp variable, and 550 bp parsimony informative sites. Computed genetic variation (Supplementary Table S3) between all distinct *Dactylogyrus* species included in the analyses (excluding data generated here) was 1.11–37.11% whereas intraspecific distances of up to 5.32% were observed. The computed genetic variability between *D. dominici* and *D. teresae* was 5.13%. The topology based on 18S-ITS1-5.8S rDNA (Fig. 8) shows five major clades. Clade A consists of *D. teresae* and *D. dominici* from *Enteromius* species. Clade B, with four species collected from *Cyprinus, Carassius* and *Luciobarbus* hosts. Clade C comprises three dactylogyrids from American *Notemigonus, Nocomis* and *Rhinichthys* hosts and clade D with 15 species from *Luciobarbus, Barbus, Telestes, Leuciscus, Rutilus, Chondrostoma, Vimba, Aspius* and *Abramis* hosts. Clade E is basal to all the other *Dactylogyrus* species included in the analysis and comprises four species from *Labeobarbus* and *Carasobarbus* hosts. All included data were generated from specimens collected from cyprinids and leuciscids, with none of the other data collected from *Enteromius*.

For CO1 mtDNA, two sequences were obtained for *D. dominici* (1190 bp) and five for *D. teresae* (1227 bp). The alignment with only CO1 mtDNA data generated here for *D. dominici* and *D. teresae* was 1190 bp long, with 1006 bp conserved, 184 bp variable, and 161 bp parsimony informative sites. Computed *p-*distances and bp differences are in Supplementary Table S4. The intraspecific variation for *D. dominici* was 0.17%, whereas the intraspecific variation for *D. teresae* ranged from 0 to 2.23%. The genetic variability between *D. teresae* and *D. dominici* ranged from 13.57 to 15.13%.

## Discussion

*Dactylogyrus* species of African fishes exhibit a variety of morphological types thus representing a variety of species groups. These groups include *D. afrobarbae*-like group, *D. pseudanchoratus-*like group, *D. varicorhini-*like group*, D. carpathicus-*like group, *D. guirensis*-like group and the recently proposed *D. cyclocirrus*-like group [19, 20, 24, 48, 49]. The morphological examination of both *D. dominici* and *D. teresae* agreed with Mashego’s [36] findings that both species belong to the *Dactylogyrus afrobarbae*-like group, matching the description of this group by Paperna [48, 49]. This group has the following characteristics: anchors have a long inner root and short or vestigial outer root; the transverse bar is long and subdivided into two by a medial constriction; the MCO/cirrus is long, tubiform and coils once or twice; the distal portion of the cirrus is connected to the accessory piece, which terminates with a fixed or flexible hook; the vagina, if present is either sclerotised or fibrous, and can have dentations or spines [48, 49].

The morphology of *D. dominici* with LM showed variation between specimens for some structures (Supplementary Table S5). Initially, before confirming the identity of the study specimens (with SEM of isolated sclerites and molecular analyses), two almost identical forms were observed, differing in the structure of the transverse bar. Both these forms were previously reported from *E. paludinosus* in the Barberspan Wetland [70] as two distinct unidentified species. Using the multifaceted approach in this study, GAP slides with representatives of both forms were first studied with LM and then digested for SEM. This technique successfully revealed more detail of the transverse bar, showing that it is flexible and that the knob-like ends are only on the dorsal side of the bar, appearing different in flattened specimens depending on the orientation. DNA analysis confirmed that the two transverse bar orientations identified using LM for *D. dominici* are conspecific with no intraspecific variation. Therefore, utilizing only standard LM for identification may result in misinterpretation of structures. Light microscopy study also showed variation in the morphology of the vagina of *D. dominici* in all studied specimens. The first form was dented, with a rounded base and hair-like projections, and the second form similar in shape, lacking hair-like projections but covered in what appeared to be spikes. The interpretation of the first form of the vagina within whole mounted specimens was inconsistent due to varying orientations. Post digestion, all specimens of this form were similar when studied using SEM. The technique also revealed that the vagina is sclerotised and dented, with the hair-like extensions partially digested, suggesting that they may be semi-sclerotised. However, the second type was seen only in three specimens and no genomic characterisation or SEM observations were successful. The presence and possible distinctness of the second form need further investigation.

The morphology of all specimens identified as *D. teresae* was consistent regarding all examined structures. All the taxonomically important structures matched the description of *D. teresae* except for the number of hooks. Additionally, the original description mentioned that the vagina is sclerotised with spinous outer margins. However, the multifaceted approach allowed for the comparison of the same structure in different microscopy techniques (LM and SEM). This showed that the vagina is dented and the pattern on the dented margins may be misinterpreted for spines with LM.

The study of the morphology of the majority of African *Dactylogyrus* species has been limited to standard LM techniques, *e.g.*, [14, 36, 45, 48–50, 52, 53, 71]. Studying isolated sclerites with SEM post-digestion has added supplementary information for several monogenoid taxa over four decades [16, 25, 26, 33, 39, 40, 57, 58]. The integration of LM and SEM of isolated sclerotised structures enables comprehensive interpretation of the morphology and morphometry of taxonomically important structures [16, 17, 52]. Additionally, the measurements of sclerotised structures obtained from LM and SEM for both *D. dominici* and *D. teresae,* respectively were not significantly different, allowing direct comparison of measurements obtained with both techniques. This supports the findings in previous studies on mono- and polyopisthocotyleans such as *Gyrodactylus* species [18, 34, 46, 57, 58] *Macrogyrodactylus* species [33], *Dactylogyrus* species [46] and diplozoids [16, 17]. The present study is the first to isolate and examine the sclerotised vagina of a dactylogyrid with SEM and to use the same specimen for both LM and SEM techniques, enabling a more holistic interpretation of sclerotised structures. Attempts to genetically characterise GAP-mounted specimens were unsuccessful. The failure is likely related to the low pH from the picric acid in GAP, degrading the DNA over time.

The haplotypes for all *D. dominici* and *D. teresae* specimens studied could be directly linked to their respective morphology, supporting the conspecificity of specimens, irrespective of the observed morphological variation. These taxa are topologically closely related, supported by genetic distances, yet genetically distinct. The 28SrDNA topology showed close relation between *D. aspili, Dactylogyrus* sp. (KY629358) and the group comprising *D. dominici* and *D. teresae.* These four species parasitise *Enteromius* Cope, 1867 hosts. Three of these four species (*D. dominici*, *D. teresae* and *D. aspili*) have similar type-morphology of the sclerotised structures; however, the morphology of the *Dactylogyrus* sp. has not been reported. There is no record assigning *D. aspili* to a species group, but the morphology of sclerotised structures matches the description of the *D. afrobarbae-*like group assigned by Paperna [48, 49]. This study presents the first phylogenetic study of *Dactylogyrus* species predetermined as members of *D. afrobarbae*-like group. Thus, the 28S rDNA data supports the designation of *D. aspili* to the *D. afrobarbae-*like group, while also suggesting the monophyly of this species group based on available data. Due to the absence of 18S-ITS1-5.8S rDNA data for *D. aspili* and the *Dactylogyrus* sp., the relation of *D. dominici* and *D. teresae* to other members of the *D. afrobarbae* species group could not be provided. However, the 18S-ITS1-5.8S rDNA data supported the close relatedness of the latter species, suggesting similar trends with different markers. Regarding CO1 mtDNA, phylogenetic analyses could not be performed as there are limited data and the majority of the few available sequences are unpublished.

To conclude, the morphology of *D. dominici* and *D. teresae* are revised here, alongside a new locality record and the first genetic data for both species. The results in the present study support the presence of two genetically and morphologically distinct taxa from the same host and locality. The approach employed here was to record the genetic profile and complete morphology of the same specimen, as opposed to bisection of specimens and potential loss of significant morphological data. Additionally, this is the first study of isolated sclerotised structures using SEM for *D. dominici* and *D. teresae*, and of the vagina of any dactylogyrid. This study provided the possibility of using one specimen for both LM and SEM for detailed morphometric analyses of taxonomically important structures, even those in historic collections. Furthermore, it is recommended that all future studies generate morphologically substantiated molecular data for dactylogyrids, particularly for African species as there are limited genetic data available.

## Supplementary materials

The Supplementary materials of this article are available at https://www.parasite-journal.org/10.1051/parasite/2024077/olm *Supplementary Table S1*: Calculated significant differences (*p*-values) in the point-to-point measurements of sclerotised structures obtained using light microscopy (LM) versus scanning electron microscopy (SEM) for *D. dominici* and *D. teresae* as well as significant differences in the point-to-point measurements obtained using light microscopy for the specimens from the present study and type specimens of the respective species. *Supplementary Table S2*: Uncorrected pairwise*-*distances (%, below the diagonal) and base pair (bp, above the diagonal) differences of *Dactylogyrus* species included in the 28S rDNA analyses. Intraspecific variation is indicated in shaded cells. *Supplementary Table S3*: Uncorrected pairwise*-*distances (%, below the diagonal) and base pair (bp, above the diagonal) differences of *Dactylogyrus* species included in the 18S-ITS1-5.8S rDNA analyses. Intraspecific variation is indicated in shaded cells. *Supplementary Table S4*: Uncorrected pairwise*-*distances (%, below the diagonal) and base pair (bp, above the diagonal) differences of *Dactylogyrus dominici* Mashego, 1983 and *Dactylogyrus teresae* Mashego, 1983 based on the CO1 mtDNA analyses. Intraspecific variation is indicated in shaded cells. *Supplementary Table S5*: Light micrographs of various orientations observed for the transverse bar and vagina of *Dactylogyrus dominici*.
