# Supplementary material for: Multifaceted taxonomy of two Dactylogyrus species on Enteromius paludinosus: Integrating light microscopy, scanning electron microscopy and molecular approaches
Source: Parasite. 2025 Jan 30;32:5. doi: 10.1051/parasite/2024077 (PMC11784097; doi:10.1051/parasite/2024077)
Supplement: Supplementary file 1 — Supplementary Table S1: Calculated significant differences (p-values) in the point-to-point measurements of sclerotised structures obtained using light microscopy (LM) versus scanning electron microscopy (SEM) for D. dominici and D. teresae as well as significant differences in the point-to-point measurements obtained using light microscopy for the specimens from the present study and type specimens of the respective species. [file parasite-32-5-s1.pdf]

Supplementary Table S1: Calculated significant differences (p-values) in the point-to-point measurements of sclerotised structures obtained using light microscopy (LM) versus scanning electron microscopy (SEM) for *D. dominici* and *D. teresae* as well as significant differences in the point-to-point measurements obtained using light microscopy for the specimens from the present study and type specimens of the respective species.

	Dactylogyrus dominici		***Dactylogyrus teresae***	
	LM versus SEM	Present study versus type specimens	LM versus SEM	Present study versus type specimens
**Anchor**				
Total length	0.742	<0.001	0.460	0.100
Shaft length	0.079	0.563	0.557	0.245
Inner root length	0.267	0.797	0.313	0.399
Outer root length	0.179	0.368	0.560	0.127
Point length	0.920	0.658	0.199	0.203
**Transverse bar**				
Total width	0.099	0.307	0.117	0.829
Total length	0.071	0.196	0.883	0.352
Lateral arm length	0.646	0.901	0.197	0.582
Medial part length	0.492	0.253	0.752	0.179
**Male Copulatory Organ**				
Copulatory duct	0.391	0.620	0.143	*
Accessory piece	0.349	0.163	0.265	*
Hook length	0.078	0.290	0.211	*
**Vagina**				
Total length	*	0.171	0.225	0.364
Total width	*	0.143	0.679	0.784

* Could not be computed
