# Supplementary material for: Multifaceted taxonomy of two Dactylogyrus species on Enteromius paludinosus: Integrating light microscopy, scanning electron microscopy and molecular approaches
Source: Parasite. 2025 Jan 30;32:5. doi: 10.1051/parasite/2024077 (PMC11784097; doi:10.1051/parasite/2024077)
Supplement: Supplementary file 3 — Supplementary Table S5: Light micrographs of various orientations observed for the transverse bar and vagina of Dactylogyrus dominici. [file parasite-32-5-s3.pdf]

Supplementary Table S5: Light micrographs of various orientations observed for the transverse bar and vagina of *Dactylogyrus dominici*.

**The first orientation of the transverse bar**	**The second orientation of the transverse bar**
Figure A-D (20 µm): First orientation of transverse bar comprising two sub-median folds dividing it into a slightly raised medial part conjoined to lateral arms with rounded knob-like ends.	Figure D-F (20 µm): Second orientation of transverse bar consisting of two sub-median folds dividing it into a flattened medial part connected to lateral arms with pointed ends.
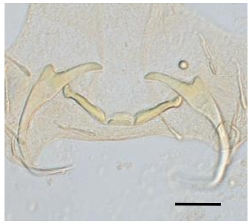 Figure A 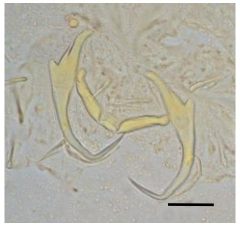 Figure B 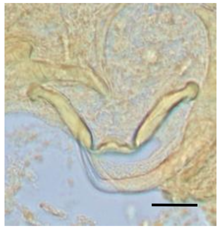 Figure C	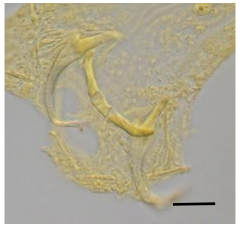 Figure D 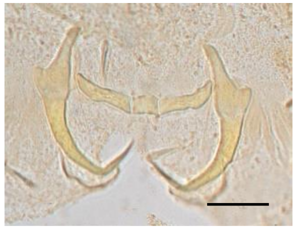 Figure E 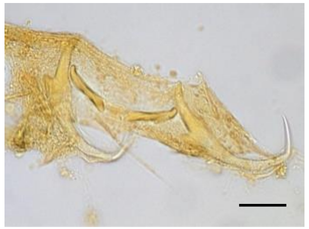 Figure F

**The first form of the vagina**	**The second form of the vagina**
Figure G-J (5 µm): First form of the vagina, with a dented base connected to a smaller part with hair-like projections. In some specimens (e.g., Figure J), the projections are replaced by a thinly lined pouch	Figure K-L (5 µm): Second form of the vagina, similar in shape but covered in what appears to be spikes.
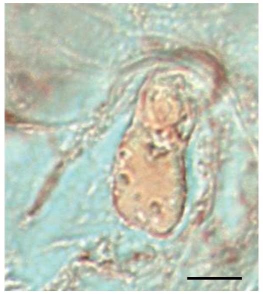 Figure G 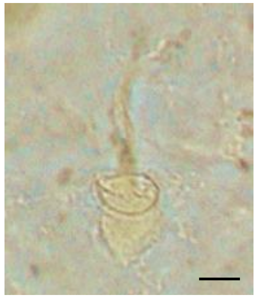 Figure H 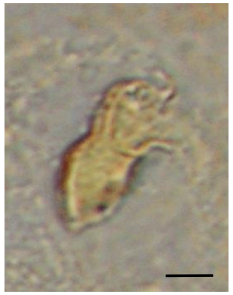 Figure I 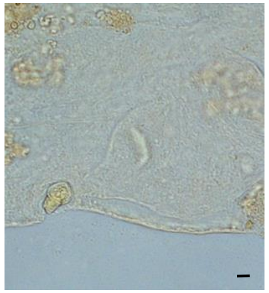 Figure J	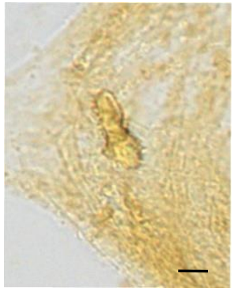 Figure K 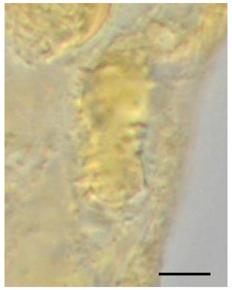 Figure L
